# Lipoprotein(a) as a Stroke Biomarker: Pathophysiological Pathways and Therapeutic Implications

**DOI:** 10.3390/jcm14092990

**Published:** 2025-04-25

**Authors:** Evangelos Panagiotopoulos, Lina Palaiodimou, Aikaterini Theodorou, Georgia Papagiannopoulou, Eleni Bakola, Maria Chondrogianni, Klearchos Psychogios, Odysseas Kargiotis, Apostolos Safouris, Charalambos Vlachopoulos, Sotirios Giannopoulos, Marios Themistocleous, Vaia Lambadiari, Georgios Tsivgoulis, Maria-Ioanna Stefanou

**Affiliations:** 1 Second Department of Neurology, “Attikon” University Hospital, School of Medicine, National and Kapodistrian University of Athens, 124 62 Athens, Greece; vaggelispana@med.uoa.gr (E.P.); katetheo24@gmail.com (A.T.); georgiapap22@hotmail.com (G.P.); elbakola@yahoo.gr (E.B.); mariachondrogianni@hotmail.gr (M.C.); apsychoyio@yahoo.gr (K.P.); safouris@yahoo.com (A.S.); sgiannop@uoi.gr (S.G.);; 2 Stroke Unit, Metropolitan Hospital, 185 47 Piraeus, Greece; kargiody@gmail.com; 3 First Department of Cardiology, Hippokration University Hospital, School of Medicine, National and Kapodistrian University of Athens, 115 27 Athens, Greece; cvlachop@otenet.gr; 4 Department of Neurosurgery, Agia Sofia Children’s Hospital, 115 27 Athens, Greece; mthemistocleous@gmail.com; 5 Research Institute and Diabetes Center, Second Department of Internal Medicine, “Attikon” University Hospital, School of Medicine, National and Kapodistrian University of Athens, 124 62 Athens, Greece; vlambad@otenet.gr; 6 Department of Neurology and Stroke, Eberhard-Karls University of Tubingen, 72074 Tubingen, Germany

**Keywords:** lipoprotein(a), stroke, atherosclerosis, small interfering RNA, antisense oligonucleotides, intracranial atherosclerosis, carotid artery diseases

## Abstract

Lipoprotein(a) [Lp(a)] has attracted widespread interest as a potential biomarker for cerebrovascular diseases due to its genetically determined and stable plasma concentration throughout life. Lp(a) exhibits pro-atherogenic and pro-thrombotic properties that contribute to vascular pathology in both extracranial and intracranial vessels. Elevated Lp(a) levels are strongly associated with large-artery atherosclerotic stroke, while data on its role in other ischemic subtypes and hemorrhagic stroke remains limited and inconsistent. Recent advances in Lp(a)-lowering therapies, such as antisense oligonucleotides and RNA-based agents, have demonstrated significant efficacy in reducing plasma Lp(a) levels. These advances have prompted increasing research into their potential application in the prevention and treatment of cerebrovascular diseases, aiming to determine whether Lp(a) reduction may translate into a reduced risk of stroke and large-artery atherosclerosis. This narrative review summarizes the current evidence on the association between Lp(a) and stroke, focusing on its utility in patient risk stratification. It also highlights existing knowledge gaps and outlines directions for future research, particularly in understanding subtype-specific effects and evaluating the clinical benefits of Lp(a)-targeted therapies.

## 1. Introduction

According to the World Stroke Organization (WSO) Global Stroke Fact Sheet 2025 and the most recent Global Burden of Disease (GBD) 2021 study, stroke remains the second leading cause of death and the third leading cause of combined death and disability worldwide [1]. Stroke recurrence is estimated to occur in approximately 9% of patients within six months after the first acute ischemic stroke (AIS) or transient ischemic attack (TIA) and in 10–16% of patients within one year, with metabolic risk factors, including hyperlipidemia, accounting for 68.8% of the global stroke burden [1,2]. Among recurrent stroke subtypes, large-artery atherosclerosis (LAA) and cardioembolic strokes are the most frequent, with recurrence rates remaining stable over the last decade [3,4]. Notably, patients with intracranial or extracranial atherosclerosis, particularly those with vulnerable plaques, severe stenosis, and poor collateral flow, remain at a sustained high risk of recurrent stroke, even when their risk factors are well-controlled [5]. These findings underscore the critical need for innovative therapeutic strategies to address atherosclerotic disease, potentially by targeting lipoprotein(a) [Lp(a)] levels.

Lp(a) has recently emerged as a key biomarker in vascular pathology, with growing evidence linking elevated plasma levels to an increased risk of AIS and TIA [6]. Individuals in the highest Lp(a) quartile may exhibit up to a 45% greater risk of ischemic stroke than those in the lowest quartile [6]. Unlike traditional lipid parameters, Lp(a) levels are largely genetically determined, remain stable throughout life, and show marked racial and interindividual variability [7]. In particular, Black individuals have nearly threefold higher median plasma Lp(a) concentrations than White individuals [8].

Structurally, Lp(a) is similar to low-density lipoprotein (LDL) but contains apolipoprotein(a) [Apo(a)], a glycoprotein that imparts pro-atherogenic, pro-inflammatory, and pro-thrombotic properties. These features are believed to contribute to ischemic stroke, predominantly through the LAA subtype [7,9]. Observational and Mendelian randomization studies have demonstrated an association between high Lp(a) concentrations and cerebrovascular events, particularly in individuals without traditional cardiovascular risk factors or stroke in the young [10,11]. Despite this growing recognition, the precise contribution of Lp(a) to different stroke subtypes—including small vessel disease, cardioembolic stroke, and embolic stroke of undetermined source (ESUS)—remains an area of active investigation [12]. Given the role of Lp(a) in vascular risk stratification, its measurement is increasingly accepted as a relatively affordable diagnostic tool for clinical implementation, particularly considering its strong predictive value for atherosclerotic cardiovascular disease (ASCVD) and stroke [13].

Despite its recognized clinical importance, there are currently no approved therapies that specifically target Lp(a) levels. Recent advances in antisense oligonucleotide (ASO) and small interfering RNA (siRNA) therapies have renewed interest in Lp(a)-lowering strategies, particularly for stroke prevention and recurrence [14]. Nevertheless, not all individuals with elevated Lp(a) levels will derive equal benefit, as stroke is a multifactorial disease influenced by a broader atherosclerotic profile, endothelial dysfunction, and pro-thrombotic factors [15]. Therefore, establishing Lp(a) thresholds for intervention remains an unmet clinical need, along with refining patient selection through comprehensive atherosclerotic risk profiling [16].

Specific populations, such as individuals with early-onset cerebrovascular disease, recurrent stroke despite optimal cardiovascular risk factor management or significant intracranial and extracranial atherosclerotic stenosis, may be the most suitable candidates for Lp(a) lowering strategies. Ongoing randomized controlled trials (RCTs) are expected to clarify patient selection strategies and define the clinical utility of emerging treatments [17,18].

Given the absence of dedicated stroke-focused trials, further research is needed to define the patient subgroups that will benefit most from these novel therapies and establish evidence-based guidelines integrating Lp(a) within the broader framework of stroke prevention and atherosclerotic risk management. This narrative review aims to provide a comprehensive evaluation of Lp(a) in stroke pathophysiology, its relevance in different stroke subtypes, and the implications of emerging therapeutics in clinical practice.

## 2. Methods

We conducted a comprehensive literature search across PubMed and Scopus up to February 2025, using combinations of the following search terms: “lipoprotein(a)”, “stroke”, “ischemic”, “hemorrhagic”, “atherosclerosis”, “intracranial” and “cerebrovascular disease”. We included peer-reviewed studies (randomized controlled trials, observational studies, narrative reviews, systematic reviews, and clinical practice guidelines) published in English that addressed the role of lipoprotein(a) in stroke. Thematically irrelevant studies, animal studies, editorials, case reports, commentaries, and preprints were excluded. Duplicate records were removed, and an initial screening based on the title and abstract was performed by three independent reviewers (EP, MIS, and GT). A multidisciplinary expert panel (CV, SG, and GT) subsequently reviewed the eligible studies and selected them for inclusion based on scientific quality, relevance, and clarity. The full search strategy and PRISMA flowchart outlining the selection process are provided in Supplementary Materials.

## 3. Review of Current Literature

### 3.1. Structure, Genetics, Epidemiology and Quantification of Lp(a)

**Figure 1:**
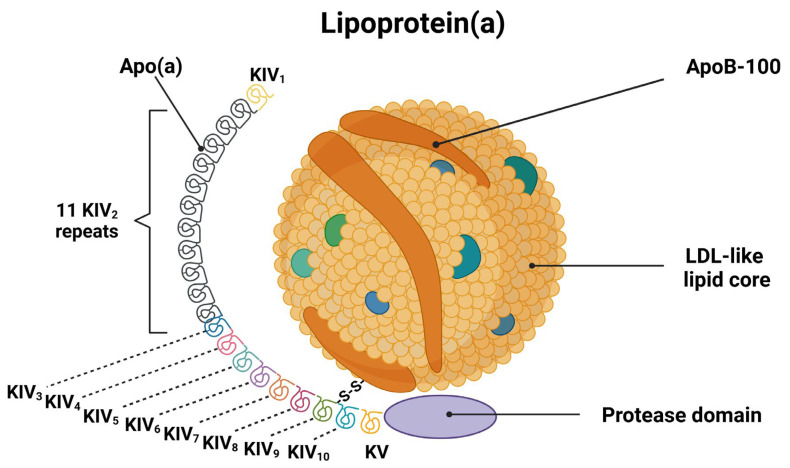
The structure of Lp(a). Lp(a) consists of an LDL-like lipid core connected to ApoB-100. A unique glycoprotein, Apo(a), is covalently attached to ApoB-100 via a disulfide bond. Apo(a) contains multiple KIV domains, including variable KIV2 repeats, single copies of KIV1 and KIV3-KIV10, and a single KV domain. Apo(a) also possesses an inactive protease domain. Variability in the KIV2 repeat number contributes to the heterogeneity of Apo(a) isoforms and influences Lp(a) plasma levels. Greater variability in KIV2 repeats leads to larger Apo(a) isoforms, which are less efficiently produced in the liver, resulting in lower Lp(a) plasma levels. Conversely, fewer repeats produce smaller isoforms that are more efficiently synthesized and secreted, leading to higher Lp(a) levels in the blood. Abbreviations: Apo(a): Apolipoprotein(a); ApoB-100: Apolipoprotein B-100; LDL: Low-density lipoprotein; KIV: Kringle IV; KV: Kringle V.

Lp(a) is a lipoprotein synthesized almost exclusively in the liver. It is structurally similar to LDL and consists of a lipid core rich in cholesteryl esters and triacylglycerols, enclosed by a phospholipid and free cholesterol shell, along with apolipoprotein B-100 (ApoB-100) [7]. However, Lp(a) has a unique structure that distinguishes it from other lipoproteins. While Lp(a) and LDL share the same lipid core and similar ApoB-100 particles, Lp(a) differs from LDL due to the presence of an additional glycoprotein, Apo(a), which is attached to ApoB-100 via a disulfide bond [7]. Apo(a) consists of multiple repeated plasminogen-like loop structures called kringle IV (KIV) domains, a single kringle V domain, and an inactive protease domain. The KIV domains are further classified into 10 distinct types (KIV1-KIV10) based on their specific amino acid sequences (Figure 1).

Notably, while most KIV domains exist as single copies, KIV2 varies in size, contributing to significant heterogeneity in Apo(a) isoforms [7]. Smaller Apo(a) isoform sizes are associated with higher Lp(a) plasma levels due to increased hepatic production [19]. In addition to ApoB-100 and Apo(a), recent studies have identified over 35 additional proteins on the Lp(a) surface, potentially contributing to its thrombogenic properties, including apolipoprotein A1 (apoA1), apolipoprotein C3 (apoC3), apolipoprotein C1 (apoC1), and apolipoprotein F (apoF) [20]. Following its secretion into the circulation, Lp(a) interacts with the vascular endothelium, contributing to inflammation and atherosclerosis, a process that likely extends to cerebral vessels, where its accumulation may promote local inflammation and atherosclerotic changes, contributing to cerebral vascular pathology [21]. Although the mechanisms of Lp(a) clearance remain incompletely elucidated, current evidence suggests that clearance cascades involve the liver (through LDL receptor-mediated uptake), kidneys, and arterial wall [22].

The structure and plasma levels of Lp(a) are primarily genetically determined and vary among populations. The LPA gene, which shares approximately 70% homology with the plasminogen gene, is located on chromosome 6 and encodes two alleles, each producing an Lp(a) isoform with distinct Apo(a) sizes [23]. Within the LPA locus, single-nucleotide polymorphisms (SNPs) can alter Apo(a) isoforms and consequently influence Lp(a) plasma concentrations [24]. Beyond the LPA locus, several genes have been proposed to regulate LPA gene expression, although their roles remain unclear [24,25].

Additional factors influencing plasma Lp(a) levels include LPA mRNA stability and translational efficiency [23]. Consequently, 70% to 90% of Lp(a) plasma levels are genetically driven and exhibit racial variation. Average Lp(a) concentrations are higher in African and South Asian populations than in East Asians and Caucasians [8,26]. Notably, data from the United Kingdom National Biobank reveal significant differences in median Lp(a) values among ethnic groups: Chinese (16 nmol/L), White (19 nmol/L), South Asian (31 nmol/L), and Black individuals (75 nmol/L) [8]. Similarly, Africans and Asians exhibit a three- to fourfold higher prevalence of intracranial atherosclerotic disease (ICAD) than Whites, suggesting a potential association between elevated Lp(a) levels and ICAD [27,28,29]. Moreover, several studies have suggested that women tend to have higher Lp(a) concentrations than men do [30,31].

The measurement of Lp(a) plasma concentrations and their clinical implications remains challenging due to the lack of standardized cutoffs, variability in influencing factors, and differences in the measurement methods. Although there is no specific guideline for an Lp(a) cutoff value, two thresholds-30 mg/dL, and 50 mg/dL, have traditionally been used to identify patients at increased risk for ASCVD [32]. The 2022 “European Atherosclerosis Society consensus statement” proposes Lp(a) cutoff values to “rule out” risk (<30 mg/dL or <75 nmol/L) and “rule in” risk (>50 mg/dL or >125 nmol/L), while values in the transitional range (30–50 mg/dL; 75–125 nmol/L) should be considered in the context of other atherosclerotic risk factors for risk stratification [16].

Although less studied, certain non-genetic factors, including dietary habits, hormonal changes, chronic kidney disease, hepatic impairment, and inflammatory conditions, may have a modest influence on Lp(a) concentrations [16]. Current guidelines recommend measuring Lp(a) in nmol/L, as this reflects the actual number of Lp(a) particles, providing a more accurate cardiovascular risk assessment than mg/dL, which measures total mass and is affected by Apo(a) isoform size [23]. While a universal conversion between mg/dL and nmol/L exists, it is not recommended due to the variability in Apo(a) isoform size [23]. Therefore, Lp(a) should ideally be measured using the same method and, when possible, within the same laboratory to ensure consistency in the longitudinal assessments [16,23].

Lp(a) levels should be carefully assessed using a combination of clinical and imaging findings. For individuals with elevated Lp(a) levels measured in nmol/L (>125 nmol/L), screening for modifiable non-genetic factors (i.e., dietary habits, hormonal changes, chronic kidney disease, hepatic impairment, and inflammatory conditions) that may increase Lp(a) is recommended, followed by consideration of Lp(a)-lowering agents as appropriate. Conversely, individuals with decreased Lp(a) concentrations (<75 nmol/L), particularly those admitted with AIS or TIA, should not be prematurely classified as ‘low-risk’. Instead, they should undergo initial neuroimaging evaluation for abnormalities such as intracranial atherosclerotic stenosis (ICAS), white matter lesions, and cerebral microbleeds, along with carotid imaging for extracranial atherosclerotic stenosis (ECAS). Such findings may still indicate Lp(a)-related vascular pathologies and warrant repeat measurements, as initial values may represent false negatives due to assay limitations.

### 3.2. Pathophysiology in Stroke

#### 3.2.1. Lipoprotein(a) and Ischemic Stroke

Lp(a) has recently gained attention as a potential biomarker for ischemic stroke prognosis. While it has long been studied as a predictor of cardiovascular disease (CVD), its association with ischemic stroke has emerged only recently [33]. Although no conclusive evidence or fully consistent data exist, the majority of observational studies across diverse populations have demonstrated a positive correlation between elevated Lp(a) plasma levels and ischemic stroke incidence, with higher concentrations nearly doubling the risk of ischemic stroke [6,34,35,36,37]. Moreover, post-hoc analyses of the Copenhagen General Population Study (CGPS) and the Copenhagen City Heart Study (CCHS), which measured plasma Lp(a) levels, along with LPA KIV type 2 number of repeats and the SNP LPA rs10455872, which represent key genetic determinants of circulating Lp(a) levels in the Danish general population, revealed that individuals with Lp(a) levels in the top 5% of the concentration distribution presented a 60% higher risk of ischemic stroke and a 17% higher absolute 10-year risk of stroke compared to those with lower Lp(a) values [37]. Similarly, findings from the Atherosclerosis Risk in Communities (ARIC) study and the Reasons for Geographic And Racial Differences in Stroke (REGARDS) cohort, which measured Lp(a) in Black and White individuals from the general population in the United States, suggested that higher Lp(a) plasma levels were associated with a 45% to 80% greater incidence of ischemic stroke, particularly in Black men and White women [6,10]. Recently, Mendelian randomization studies have utilized genetic variants that are randomly inherited and unaffected by environmental factors to infer a causal relationship between elevated Lp(a) levels and ischemic stroke, avoiding confounding factors and reverse causation that often affect observational studies [11]. These studies suggest that genetically determined elevated Lp(a) levels contribute to an increased risk of ischemic stroke and LAA stroke [11].

#### 3.2.2. Lipoprotein(a) and Ischemic Stroke Subtypes

The association between Lp(a) and the incidence of different ischemic stroke subtypes, classified according to the TOAST (Trial of Org 10172 in Acute Stroke Treatment) criteria, has been previously investigated [12]. A recent meta-analysis assessing the correlation between Lp(a) levels and the risk of stroke demonstrated that elevated Lp(a) levels were associated with an increased risk of ischemic stroke [standardized mean difference (SMD), 0.76; 95% CI, 0.53–0.99], particularly in the LAA subtype (SMD, 0.68; 95% CI, 0.01–1.34) [12]. However, the available data remain limited, and further research is required to establish a definitive correlation between plasma Lp(a) levels and specific stroke subtypes (Table 1). The potential pathophysiological mechanisms underlying each stroke subtype are discussed in the following sections.

**Table 1:** Stroke subtypes and potential associations with Lp(a).

Stroke Type	Potential Association with Lp(a)
LAA	Elevated Lp(a) is associated with an increased risk of LAA [9].
CE	Elevated Lp(a) is associated with a moderate risk of CE [38].
cSVD	Contradictory findings regarding the association of Lp(a) with cSVD; elevated Lp(a) potentially associated with reduced cSVD risk [39].
Cryptogenic/ESUS	Elevated Lp(a) is associated with a moderate risk of cryptogenic stroke/ESUS [40].
ICH	Contradictory findings regarding the association of Lp(a) with ICH; elevated Lp(a) potentially associated with reduced ICH risk [41].
ICH-CAA	Elevated Lp(a) is associated with potentially protective effects in CAA, based on limited evidence [42].
SAH	Elevated Lp(a) is associated with a moderate risk of SAH, based on preliminary findings [43].

Abbreviations List: LAA: Large-Artery Atherosclerosis; CE: Cardioembolic Stroke; cSVD: Cerebral Small Vessel Disease; ESUS: Embolic Stroke of Undetermined Source; ICH: Intracerebral Hemorrhage; CAA: Cerebral Amyloid Angiopathy; SAH: Subarachnoid Hemorrhage; Lp(a): Lipoprotein(a).

##### Lipoprotein(a) and Large-Artery-Atherosclerosis

**Figure 2:**
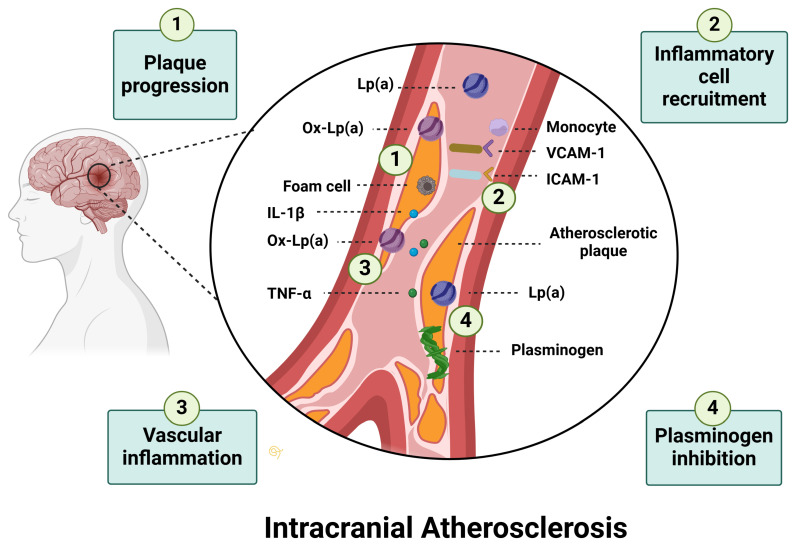
Pathophysiology of Lp(a)-induced intracranial atherosclerosis. Lp(a) contributes to atherosclerosis in large intracranial vessels through several mechanisms. (1) Plaque progression: Lp(a) promotes smooth muscle cell proliferation by inhibiting the conversion of plasminogen to plasmin, which prevents plasmin-mediated activation of TGF-β, an autocrine inhibitor of smooth muscle cell growth. Additionally, Lp(a) facilitates foam cell formation and calcification, accelerating plaque progression. Ox-Lp(a) delivers excess OxPLs to the arterial wall, driving inflammation and plaque instability. (2) Inflammatory cell recruitment: Lp(a) upregulates adhesion molecules such as ICAM-1 and VCAM-1, facilitating the adhesion and infiltration of monocytes and macrophages into the vascular wall, further amplifying local inflammation. (3) Vascular inflammation: Lp(a) carries OxPLs, which promote endothelial dysfunction and trigger the release of inflammatory cytokines, such as IL-1β and TNF-α. (4) Plasminogen inhibition: The structural similarity between Apo(a) and plasminogen allows Lp(a) to competitively inhibit plasminogen activation, impairing fibrinolysis and increasing thrombogenicity. Abbreviations: Lp(a): Lipoprotein(a); OxPLs: Oxidized phospholipids; Ox-Lp(a): Oxidized lipoprotein(a); IL-1β: Interleukin-1 beta; TNF-α: Tumor necrosis factor-alpha; ICAM-1: Intracellular adhesion molecule-1; VCAM-1: Vascular cell adhesion molecule-1.

Among ischemic stroke subtypes, LAA has the strongest association with Lp(a) plasma levels. Previous studies have demonstrated that increased Lp(a) levels contribute to ICAD [9,39,44]. In patients admitted with AIS, Lp(a) levels are significantly higher in those with LAA stroke than in those with non-LAA stroke subtypes [odds ratio (OR): 2.4; 95% CI: 1.39–3.93; *p* = 0.001] [44]. Several mechanisms have been proposed through which Lp(a) induces atherosclerosis in large intracranial vessels (Figure 2): (i) Pro-atherogenic effects: Lp(a)-associated oxidized phospholipids (OxPLs), derived from its LDL-like core, promote vascular inflammation and atherogenesis [7]; (ii) Pro-thrombotic properties: The structural similarity of Apo(a) to plasminogen enables Lp(a) to competitively inhibit plasminogen activation, impairing fibrinolysis and increasing the risk of clot formation within vascular plaques [45]; (iii) Inflammatory cell recruitment: Lp(a) upregulates adhesion molecules such as intracellular adhesion molecule-1 (ICAM-1) and vascular cell adhesion molecule-1 (VCAM-1), by inhibiting transforming growth factor-β (TGF-β), thereby driving monocyte/macrophage recruitment [46]; and (iv) Plaque development and calcification: Lp(a) promotes smooth muscle cell proliferation, foam cell formation, and calcification, accelerating plaque progression [7].

In addition to its critical role in intracranial atherosclerosis, Lp(a) may also contribute to the development of atherosclerosis in extracranial vessels, although there is limited evidence directly comparing its impact on intracranial and extracranial atherosclerotic lesions. One study suggested that moderately elevated serum Lp(a) levels [i.e., the third Lp(a) quartile (OR: 3.33, 95% CI:1.77–6.37 for the intracranial stenosis group, and OR: 4.82, 95% CI: 1.96–11.88 for the extracranial carotid stenosis group)] were associated with isolated intracranial and extracranial carotid stenosis, while greatly elevated serum Lp(a) levels [i.e., the fourth Lp(a) quartile (OR: 4.98, 95% CI: 1.92–12.91 for the combined intracranial and extracranial stenosis group)] were related to combined intracranial and extracranial carotid lesions [47].

While both intracranial and extracranial vessels are affected by Lp(a), their distinct anatomical features may influence the progression and complications of atherosclerosis. Intracranial arteries, characterized by thinner media, reduced elastic fibers, and minimal adventitia, tend to develop more stable atherosclerotic plaques with fewer fatty streaks and complex lesions [48,49]. In contrast, extracranial arteries, such as the carotid arteries, tend to develop plaques that are more lipid-rich and prone to embolization [48]. Moreover, prior studies have identified Lp(a) as an independent risk factor for carotid atherosclerosis (i.e., common carotid, carotid bifurcation, and internal carotid) in patients with AIS, and its levels correlate with the degree of carotid artery stenosis [50,51]. Furthermore, Lp(a) concentrations are independently related to higher intraplaque neovascularization in patients with carotid stenosis, thereby increasing plaque susceptibility to hemorrhage and rupture [52]. Intraplaque hemorrhage has been consistently shown to correlate with a high risk of artery-to-artery embolism and recurrent cerebral infarction in patients with symptomatic extracranial carotid artery disease [53]. These findings highlight Lp(a) as a critical factor in cerebrovascular atherosclerosis, warranting further research to clarify its specific contributions at different vascular sites.

In summary, the relationship between Lp(a) and LAA is supported by consistent clinical and biological evidence, with elevated levels observed more frequently in patients with intracranial and extracranial stenosis than in those without significant atherosclerotic lesions. While observational studies and Mendelian randomization analyses support a causal role, further prospective data are needed to determine whether Lp(a) primarily serves as a biomarker of atherosclerotic burden or as a modifiable therapeutic target. Stratification by ethnicity, comorbid risk factors, and plaque phenotype may help refine the interpretation of Lp(a) levels in clinical practice.

##### Lipoprotein(a) and Small Vessel Disease

In contrast to LAA strokes, Lp(a) concentrations appear to be inversely associated with cerebral small vessel disease (cSVD)-related stroke. Intriguingly, recent studies have suggested a potential protective effect of increased Lp(a) levels on cSVD [39,54,55]. In particular, a community-based study of 3059 subjects demonstrated that individuals in the highest Lp(a) tertile had a 25.9% prevalence of cSVD compared to 31.7% in the lowest tertile, with an adjusted OR (aOR) of 0.74 (95% CI: 0.60–0.92, *p* < 0.05), indicating a protective effect [39]. Additionally, in an Alzheimer’s cohort of 111 patients, participants in the highest Lp(a) tertile had a 66.2% prevalence of cSVD, compared to 94.6% in the lowest tertile. The aORs ranged from 0.109 to 0.576 (*p* < 0.05), depending on the severity of cSVD, further supporting the inverse association between Lp(a) levels and cSVD burden [42]. Furthermore, Mendelian randomization analysis has confirmed a causal association between genetically elevated Lp(a) and a reduced risk of small vessel stroke (OR: 0.92, 95% CI: 0.88–0.97, *p* = 0.001) [54]. Finally, a study investigating lipid abnormalities in 202 patients with AIS found that Lp(a) levels were twice as elevated in large-artery stroke than in lacunar stroke, suggesting that Lp(a) preferentially promotes macrovascular atherosclerosis rather than small vessel pathology [55]. Collectively, these findings suggest that elevated Lp(a) levels may reduce cSVD risk.

However, the mechanisms underlying this inverse correlation are unclear. One hypothesis proposes that Lp(a) may cross an impaired blood-brain barrier and interact with cerebrospinal fluid (CSF), where it can bind to OxPLs, potentially reducing inflammation by facilitating the recycling of oxidized lipids [56]. Additionally, Lp(a), similar to other lipoproteins such as apoA1, may regulate cholesterol metabolism within the CSF and support the structural integrity of the glioneurovascular unit [57]. The Apo(a) component of Lp(a) has also been implicated in the modification of the metabolism of apolipoprotein E (apoE) isoforms, which are essential for lipid transport and neuronal survival. This interaction may enhance cholesterol turnover and improve the function of small brain vessels [58]. Furthermore, Lp(a) shares properties with LDL cholesterol, which has been linked to reduced white matter hyperintensity (WMH) volumes on brain magnetic resonance imaging(MRI), suggesting a possible protective effect on brain microvascular structures [59]. Notably, recent evidence suggests that higher Lp(a) concentrations are associated with lower volumes of WMH, cerebral microbleeds, basal ganglia-enlarged perivascular spaces, and lacunes [39,42].

In contrast, a recent study reported contradictory findings, suggesting that elevated Lp(a) levels may contribute to white matter lesions, potentially counteracting its beneficial effects on cSVD. This study, which included 153 patients with AIS or TIA, indicated that those with Lp(a) levels greater than 125 nmol/L exhibited a nearly 5-fold higher burden of both periventricular WMH (95% CI: 1.60–12.07, *p* = 0.004) and deep WMH (95% CI: 1.69–14.7, *p* = 0.001) than those with Lp(a) levels lower than 75 nmol/L [60]. Further research is needed to clarify and validate these complex correlations.

Taken together, most available evidence suggests an inverse relationship between elevated Lp(a) levels and stroke in patients with cSVD. This association is primarily supported by multicenter, well-characterized cohorts employing predefined protocols and robust statistical analyses [39] with large sample sizes [54]. In contrast, the only study reporting a positive association was based on a small cohort of patients (*n* = 153) presenting with acute lacunar stroke and was constrained by a retrospective observational design, limiting the strength and generalizability of its findings [60]. These contrasting findings underscore the need for large, multicenter prospective studies with standardized imaging protocols and consistent Lp(a) assessments to clarify its role in small vessel cerebrovascular pathology.

##### Lipoprotein(a) and Cardioembolic Strokes

The correlation between serum Lp(a) levels and the incidence of cardioembolic stroke is unclear. The association between Lp(a) and atrial fibrillation (AF), a significant contributor to cardioembolic stroke, has been the subject of ongoing scientific investigation. Prior research has concluded that there is no relationship between elevated Lp(a) plasma levels and the risk of AF or cardioembolic stroke [12,61]. Likewise, the 2022 “European Atherosclerosis Society consensus statement” did not provide evidence establishing a direct association between Lp(a) levels and the risk of AF or cardioembolic strokes [16].

However, recent studies utilizing Mendelian randomization analyses suggest that a 50 nmol/L increase in Lp(a) levels is associated with a heightened risk of developing AF, observed in both directly measured Lp(a) levels and genetically predicted Lp(a) levels [38,62]. Moreover, elevated Lp(a) levels are recognized as risk factors for calcific aortic valve disease (CAVD) and aortic valve stenosis [16,63]. Severe aortic valve disease can lead to left atrial dilation and increase the risk of thrombus formation and AF, potentially contributing to cardioembolic stroke [64]. A recent study on patients with non-valvular atrial fibrillation stratified by Lp(a) quartiles demonstrated that those in the highest Lp(a) quartile had a 2.38-fold increased risk of systemic embolism compared to those in the lowest quartile [65]. Additionally, another study demonstrated that Lp(a) levels exceeding 50 mg/dL may contribute to myocardial fibrosis and structural cardiac remodeling, including atrial dilation and left ventricular dysfunction, which increases the risk of atrial fibrillation and thrombus formation [66]. These cardiac changes may predispose individuals to cardioembolic stroke; however, further research is needed to confirm these correlations. As a result, while direct evidence linking Lp(a) to cardioembolic stroke is limited, its role in atrial remodeling and valvular disease may contribute indirectly.

##### Lipoprotein(a) and Strokes of Undetermined Etiology

Although Lp(a)’s causal relationship with cryptogenic strokes and ESUS is the least examined among the aforementioned stroke subtypes, it is of significant scientific interest, as these stroke subtypes account for over 40% of ischemic strokes in young patients [67]. Three potential pathophysiologic mechanisms have been proposed to explain Lp(a)’s contribution to cryptogenic and ESUS strokes. First, elevated serum Lp(a) concentrations can destabilize non-stenotic atherosclerotic plaques in the intracranial and extracranial carotid arteries, which often evade detection by conventional neuroimaging, leading to embolization in distal arteries and subsequent ischemic infarcts [40,68,69,70,71]. Additionally, vulnerable atherosclerotic plaques in the aortic arch (aortic arch atheroma) represent another potential source of unrecognized embolism [7,72,73]. Second, prior research suggests a potential correlation between elevated Lp(a) levels and venous thromboembolism, although this association remains uncertain [16,74]. Taking the foregoing into account, elevated Lp(a) levels may promote a pro-thrombotic environment, resulting in paradoxical embolism in patients with patent foramen ovale (PFO) [16,75]. Third, Lp(a)’s role in thrombogenicity may be mediated through several additional mechanisms, including platelet activation and aggregation, interaction with tissue factor (TF), inhibition of fibrinolysis by plasmin, and increased expression of plasminogen activator inhibitor-1 (PAI-1), which collectively facilitate thrombus formation in intracranial and extracranial vessels, potentially leading to cerebral infarction [7,65]. The limited evidence regarding Lp(a)’s association with strokes of undetermined etiology, particularly in young patients, highlights a promising avenue for future research [76,77].

#### 3.2.3. Lipoprotein(a) and Hemorrhagic Stroke

Notwithstanding the widely accepted role of Lp(a) in atherogenesis, its involvement in hemorrhagic stroke remains a topic of ongoing debate. Two studies conducted in the Chinese population, along with a meta-analysis, reported an association between elevated Lp(a) plasma levels and an increased risk of intracerebral hemorrhage (ICH) [12,78,79]. The first study demonstrated that individuals in quartiles 2, 3, and 4 had 1.93-fold, 3.24-fold, and 2.19-fold increased odds of ICH, respectively, compared to those in quartile 1 (*p* < 0.05) [78]. The second study reported an OR of 1.64 (95% CI: 1.21–2.21) for elevated Lp(a) levels [79]. Additionally, a meta-analysis that compared patients admitted with ICH to controls identified an SMD of 0.65 (95% CI: 0.13–1.17), further supporting this association [12]. Conversely, other studies have demonstrated an inverse association between elevated Lp(a) concentrations and the incidence of hemorrhagic stroke [41], while some have detected no association [34,80].

To determine whether a definite connection between Lp(a) and hemorrhagic strokes exists, it is crucial to investigate the potential mechanisms underlying Lp(a)’s role in ICH. Elevated Lp(a) levels may exert a protective effect in ICH associated with cerebral amyloid angiopathy (ICH-CAA) and cerebral microbleeds through interactions with apoE [42]. Lp(a) has been implicated in modulating apoE metabolism by forming apoE-enriched Lp(a) particles, which accelerate apoE turnover and enhance cholesterol clearance, potentially stabilizing amyloid-laden vessels and reducing vascular fragility [58]. By binding to or competing with apoE receptors, Lp(a) may mitigate the effects of apoE4, a recognized risk factor for CAA, thereby limiting amyloid deposition and improving vascular integrity [81]. Conversely, lower Lp(a) levels may impair these protective processes, leading to increased amyloid accumulation, greater vessel fragility, and a higher risk of ICH-CAA and microbleeds [42,82]. Similarly, Lp(a)’s ability to inhibit plasminogen activation and fibrinolysis can lead to clot stabilization, reducing the risk of vessel rupture in patients with CAA [7]. Given these potential protective effects, screening for Lp(a) levels in patients with CAA could be considered a means of stratifying bleeding risk and identifying those who may be at higher risk of ICH.

Beyond its potential role in CAA-related hemorrhages, Lp(a) has also been implicated in the pathogenesis of intracranial aneurysms and their subsequent risk of rupture. Evidence suggests that elevated plasma Lp(a) levels are associated with increased concentrations of Lp(a) within intracranial aneurysm sacs, where they may contribute to aneurysm wall inflammation and instability [43,83]. This inflammatory response may weaken the vascular wall, increasing susceptibility to aneurysm formation and rupture, ultimately leading to ICH.

Overall, the discordant findings regarding Lp(a) and hemorrhagic stroke likely reflect heterogeneity in study design [78] and etiological classification of ICH [41]. In terms of underlying mechanisms, increased Lp(a) concentrations have been associated with a greater risk of aneurysmal ICH [43], whereas an inverse association has been observed in cases of ICH-CAA and cerebral microbleeds [42]. These discrepancies highlight the need for future studies employing standardized methodologies and rigorous etiological stratification to better clarify the role of Lp(a) in hemorrhagic stroke.

#### 3.2.4. Lipoprotein(a), Post-Stroke Recovery and Stroke Recurrence

Lp(a)’s pro-atherogenic, pro-thrombotic, and pro-inflammatory profile may adversely affect both recovery and the risk of recurrence in patients with AIS. Previous research has underscored the association between elevated Lp(a) levels and worse long-term outcomes after stroke [62,84,85]. A recent study demonstrated that patients with increased Lp(a) plasma levels combined with low LDL levels presented a greater risk of major disability and death at 6 months compared to those with aligned Lp(a) and LDL levels (aOR: 1.59; 95% CI: 1.01–2.52) [86]. Furthermore, Lp(a) serum values exceeding 30 mg/dL have been correlated with a 2.6-fold increased risk of cerebrovascular events in patients with AIS, including recurrent ischemic stroke and TIA at 12-month follow-up (95% CI: 1.19–5.67; *p* = 0.016) [87]. In addition, Lp(a) concentrations exceeding 50 mg/dL in patients admitted with AIS or TIA were associated with a significantly higher risk of stroke recurrence at 1-year follow-up compared to those with baseline Lp(a) values below 50 mg/dL (11.5% versus 9.4%; adjusted HR: 1.20; 95% CI: 1.02–1.42) [88].

Elevated Lp(a) levels may also pose challenges in the management of AIS. As previously demonstrated, the pro-atherogenic properties of Lp(a) play a critical role in the development and progression of ICAD and LAA strokes [47,89]. A recent study involving 553 patients with AIS who underwent percutaneous transluminal angioplasty and stenting (PTAS) for secondary stroke prevention due to ICAS and ECAS indicated that Lp(a) plasma levels higher than 30 mg/dL were an independent risk factor for recurrent ischemic stroke in symptomatic patients with intracranial and extracranial artery PTAS [90]. Similarly, Lp(a) levels have been related to increased plaque vulnerability in in-stent neoatherosclerosis [91]. Additionally, among 236 patients with AIS who received thrombolysis, Lp(a) was identified as an independent risk factor for early neurological deterioration, particularly when its levels exceeded 300 mg/L (OR: 3.154; 95% CI: 1.067–9.322; *p* = 0.038) [92]. Consequently, Lp(a)’s atherosclerotic profile may indirectly influence the effectiveness of EVT in AIS by contributing to more complex vascular pathology, higher complication rates, and worse outcomes.

### 3.3. Therapies Targeting Lipoprotein(a)

To date, no specific Lp(a)-lowering drugs have been approved for directly addressing elevated Lp(a) levels, and current guidelines focus solely on Lp(a) measurement and screening. In accordance with the recent European Society of Cardiology (ESC)/European Atherosclerosis Society (EAS) and the Canadian Cardiovascular Society (CCS) guidelines for the management of dyslipidemias, Lp(a) should be measured at least once in adulthood to identify individuals at increased atherosclerotic risk [93,94]. Screening for Lp(a) is also suggested for young individuals with a history of ischemic stroke, a family history of premature ASCVD, or elevated Lp(a) levels, especially in the absence of other identifiable risk factors [16].

#### 3.3.1. Existing Strategies

Despite the current absence of clinically approved pharmacotherapies specifically targeting elevated Lp(a) plasma levels, existing therapeutic agents have been shown to influence Lp(a) concentrations. Statins, which are widely utilized for hypercholesterolemia management and secondary stroke prevention, have been associated with potential increases in Lp(a) levels [95,96]. In a prospective cohort study of patients with first-ever AIS, nearly half of the statin-treated individuals exhibited elevated Lp(a) levels during follow-up compared to baseline, which correlated with recurrent vascular events despite achieving LDL cholesterol targets [97]. Nevertheless, the latest “European Atherosclerosis Society Consensus Statement” continues to advocate statin therapy, emphasizing that its cardioprotective benefits outweigh the potential risks associated with Lp(a) elevation [16].

In contrast, proprotein convertase subtilisin/kexin type 9 inhibitors (PCSK9i), such as alirocumab, evolocumab, and inclisiran, have demonstrated the ability to reduce Lp(a) serum levels by 25–30% and concurrently decrease the risk of stroke [14,98,99,100]. However, whether these benefits are mediated directly by Lp(a) reduction or through the coexisting reduction of LDL cholesterol remains unclear [62]. Lipoprotein apheresis, approved by the Food and Drug Administration (FDA) as an Lp(a)-lowering therapy for patients with plasma Lp(a) levels exceeding 60 mg/dL, offers an alternative approach, although evidence of its impact on overall atherosclerotic risk remains limited [23]. Furthermore, niacin (nicotinic acid) has also been explored as an Lp(a)-lowering agent, reducing levels by 20–30% [101]. However, niacin is not currently approved, as this reduction in Lp(a) has not been associated with any clinical benefits in atherosclerotic events [7,102]. Finally, the possible correlation between aspirin and Lp(a) levels, as well as their impact on the incidence of atherosclerotic ischemic events, remains incompletely understood [16,103]. Further research is needed to determine whether existing pharmacotherapies can be successfully adapted for Lp(a) management.

#### 3.3.2. Emerging Therapies

ASOs represent a promising alternative for addressing elevated levels of Lp(a). ASOs are synthetic, single-stranded oligodeoxynucleotides able to interrupt Lp(a)’s hepatic production by directly degrading mRNA via ribonuclease H or by blocking translation, thereby preventing the production of Apo(a) [104]. Pelacarsen (formerly TQJ230 and AKCEA-APO(a)-LRx), a second-generation ASO, has demonstrated significant dose-dependent efficacy, achieving reductions in mean Lp(a) concentrations of 30–90% across several phase 1 and 2 RCTs [105]. The ongoing HORIZON (Assessing the Impact of Lipoprotein(a) Lowering with TQJ230 on Major Cardiovascular Events in Patients with CVD) trial (NCT04023552) is the first phase 3 RCT evaluating the effect of monthly subcutaneous administration of pelacarsen (80 mg) in reducing major adverse cardiovascular events (MACE) (i.e., cardiovascular death, non-fatal myocardial infarction, non-fatal stroke, and urgent coronary revascularization requiring hospitalization) in patients with Lp(a) plasma levels exceeding 70 mg/dL and a history of myocardial infarction, ischemic stroke, or peripheral arterial disease (Table 2) [17]. Scheduled for completion in May 2025, this trial will provide critical insights into whether Lp(a) lowering translates into reduced stroke incidence and overall atherosclerotic risk [106].

In contrast to ASOs, siRNA therapies use double-stranded RNA to target the LPA gene by degrading LPA mRNA through the RNA interference (RNAi) pathway prior to translation, thereby preventing the synthesis of Apo(a) and reducing Lp(a) plasma levels [107]. Olpasiran (formerly AMG 890), a liver-directed siRNA that specifically targets LPA mRNA, reduces Apo(a) production and, like pelacarsen, is administered subcutaneously, with long-acting effects allowing dosing intervals of several months [14]. Phase 1 and 2 RCTs have shown that olpasiran lowers Lp(a) levels by up to 90% in patients with elevated Lp(a) concentrations [108,109]. The ongoing OCEAN(a) (Olpasiran Trials of Cardiovascular Events and Lipoprotein(a) Reduction)-Outcomes trial (NCT05581303) is a phase 3 RCT evaluating the efficacy and safety of olpasiran in reducing the risk of MACE (i.e., coronary heart disease death, myocardial infarction, or urgent coronary revascularization) in patients with ASCVD and Lp(a) levels above 200 nmol/L (Table 2) [18]. Although the trial solely focused on cardiovascular events, its findings could also have implications for stroke prevention in patients with elevated Lp(a) levels. Furthermore, Zerlasiran (formerly SLN360) and Lepodisiran (formerly LY3819469) are investigational siRNAs that have shown favorable dose-dependent reductions in Lp(a) levels of up to 98% in phase 1 RCTs [110,111]. A recently published phase 2 RCT indicated that Zerlasiran reduced Lp(a) levels by more than 80% in patients with ASCVD (Table 2) [112]. Consequently, these agents hold potential as Lp(a)-lowering strategies for patients with stroke and generalized atherosclerosis. In conclusion, while no clinical data have yet confirmed a reduction in MACE or cerebrovascular events with these novel agents, their potent and sustained Lp(a)-lowering effects highlight their potential for cardiovascular and cerebrovascular risk reduction.

Unlike ASOs and siRNAs, which target mRNA, small-molecule inhibitors represent another emerging therapeutic approach that acts on proteins to prevent Lp(a) particle formation by disrupting the interaction between Apo(a) and ApoB-100 [14]. Muvalaplin, a first-in-class small-molecule inhibitor of Lp(a), is the only available oral agent dedicated to Lp(a) lowering, potentially improving patient compliance [113]. Phase 2 RCTs have shown that muvalaplin reduces Lp(a) levels by 45–85% in a dose-dependent manner (Table 2) [114]. Larger-scale RCTs are required to determine whether these reductions translate into clinical benefits for atherosclerotic events, such as stroke.

Finally, gene-editing therapies hold promise as a one-time intravenous infusion for individuals with markedly elevated Lp(a) plasma levels. Unlike siRNA and ASOs, which transiently reduce Lp(a) levels by degrading mRNA, gene editing offers a potentially permanent reduction in Lp(a) levels by disrupting or silencing the LPA gene at the DNA level [14]. Clustered regularly interspaced short palindromic repeats/CRISPR-associated protein 9 (CRISPR-Cas9) is a widely used gene-editing system that, when delivered via an adeno-associated virus (AAV) vector, can target the liver and permanently disrupt the LPA gene [14,115]. Preclinical studies using AAV-CRISPR technology in non-human primates have demonstrated high efficacy in nearly eliminating Apo(a) from circulation and achieving a sustained reduction in Lp(a) levels (Table 2) [116]. However, further research in humans is required to establish its therapeutic potential in atherosclerotic disease and rigorously assess the risk of off-target effects, including impaired cellular viability and tumorigenesis [116,117].

**Table 2:** Emerging therapies for Lp(a) reduction.

Therapy Type	Therapy Name	Mechanism of Action	Administration Route	Reduction in Lp(a) Levels	Preclinical or Clinical Trial Phase	Research Question/Findings
Antisense Oligonucleotides (ASOs)	Pelacarsen (TQJ230, AKCEA-APO(a)-LRx)	Binds to mRNA and prevents Apo(a) production	Subcutaneous	30–90%	Phase 3 ongoing (HORIZON trial, NCT04023552) [17]	Whether pelacarsen can reduce the risk of cardiovascular death, non-fatal myocardial infarction, non-fatal stroke, and urgent coronary revascularization requiring hospitalization
Small Interfering RNA (siRNA)	Olpasiran (AMG 890)	Degrades LPA mRNA to prevent Apo(a) synthesis	Subcutaneous	Up to 90%	Phase 3 ongoing (OCEAN(a)-Outcomes trial, NCT05581303) [18]	Whether olpasiran can reduce the risk of coronary heart disease death, myocardial infarction, or urgent coronary revascularization
Small Interfering RNA (siRNA)	Zerlasiran (SLN360)	Degrades LPA mRNA to prevent Apo(a) synthesis	Subcutaneous	Up to 98%	Phase 2 completed (NCT05537571) [112]	Zerlasiran can reduce Lp(a) levels by more than 80% in patients with ASCVD
Small Interfering RNA (siRNA)	Lepodisiran (LY3819469)	Degrades LPA mRNA to prevent Apo(a) synthesis	Subcutaneous	Up to 98%	Phase 1 completed (NCT04914546) [111]	Lepodisiran can be safely tolerated and lead to sustained dose-dependent Lp(a) reductions
Small-Molecule Inhibitors	Muvalaplin	Prevents Apo(a) and ApoB-100 interaction	Oral	45–85%	Phase 2 completed (NCT05563246) [114]	Muvalaplin can reduce Lp(a) levels, but the effects on cardiovascular risk remain unknown
Gene Editing	CRISPR-Cas9	Permanently disrupts the LPA gene	Intravenous	Near elimination in preclinical studies	Preclinical (Non-human primates) [116]	CRISPR-Cas9 can be safe and effective in primates

Abbreviations List: ASOs: Antisense Oligonucleotides; siRNA: Small Interfering RNA; Lp(a): Lipoprotein(a); Apo(a): Apolipoprotein(a); ApoB-100: Apolipoprotein B-100; RCT: Randomized Controlled Clinical Trial; ASCVD: Atherosclerotic Cardiovascular Disease; AAV: Adeno-Associated Virus; CRISPR-Cas9: Clustered Regularly Interspaced Short Palindromic Repeats–CRISPR-Associated Protein 9.

### 3.4. Clinical Applications and Guidance

Thus far, in the absence of specific Lp(a)-lowering therapies, guidelines for managing patients with elevated Lp(a) levels in clinical practice are limited. Emerging evidence indicates that Lp(a) levels correlate linearly with atherosclerotic risk, with the clinical benefits of Lp(a) reduction expected to be proportional to the absolute decrease in its concentration [118]. Moreover, although a threshold of 50 mg/dL has been proposed as a risk enhancer for ASCVD over a 10-year period, the “European Atherosclerosis Society consensus statement” emphasizes a continuous relationship between Lp(a) concentrations and ASCVD risk, rather than a strict cutoff [16].

Consequently, clinicians should interpret Lp(a) levels in the context of a patient’s overall ASCVD risk rather than relying on an isolated value. Notably, while elevated Lp(a) levels are associated with a greater risk of ASCVD events in high-risk individuals, some patients with markedly increased Lp(a) levels but no other major risk factors may never develop ASCVD [119]. Current ESC/EAS and CCS guidelines recommend a comprehensive approach to LDL-cholesterol management (according to current LDL-cholesterol management guidelines) and risk factor modification, including blood pressure, glucose, and lifestyle factors, for individuals with elevated Lp(a) levels [93,94]. In primary prevention, the National Lipid Association (NLA) suggests that in adults aged 40–75 years with a 10-year ASCVD risk of 7.5–19.9%, Lp(a) > 50 mg/dL may serve as a risk-enhancing factor, supporting the initiation of moderate- to high-intensity statin therapy [120]. Additionally, lipoprotein apheresis may be considered in patients with elevated Lp(a) levels and progressive atherosclerotic disease despite optimal risk factor control [16]. Ultimately, the ongoing HORIZON and OCEAN(a)-Outcomes RCTs will determine the exact degree of Lp(a) reduction required for primary and secondary stroke prevention and whether a definitive therapeutic threshold exists [17,18].

## 4. Limitations and Future Directions

Despite the growing interest in Lp(a) as a contributor to cerebrovascular disease, several important limitations in the existing literature constrain the ability to draw definitive conclusions. A substantial proportion of studies are observational, with methodological heterogeneity in design, stroke subtype classification, and endpoint definition. Inconsistencies in the literature, such as those seen in the cSVD and hemorrhagic stroke sections, likely reflect these design differences and variability in study populations and clinical settings.

Another significant challenge is the lack of standardization in Lp(a) measurements. Units (mg/dL vs. nmol/L) are often used interchangeably despite differences in what they reflect (mass vs. particle count), and cutoff values for elevated Lp(a) levels vary widely across studies. Furthermore, many reports originate from specific ethnic populations, particularly Asian cohorts [78,79], limiting their extrapolation to more diverse or Western populations. Finally, interventional data remain limited, largely due to the current absence of approved Lp(a)-lowering agents with proven efficacy in reducing stroke recurrence.

Future research should prioritize large, multi-ethnic prospective cohorts with standardized assay methods and stratification of stroke subtypes. The results of ongoing trials, such as HORIZON and OCEAN(a)-Outcomes [17,18], will be crucial in determining whether Lp(a) lowering translates into meaningful cerebrovascular benefits and in identifying the thresholds that warrant intervention in clinical neurology.

## 5. Conclusions

This narrative review provides a comprehensive synthesis of the latest evidence on Lp(a) and stroke, highlighting its evolving role as a biomarker for cerebrovascular disease, particularly in LAA. While Lp(a) has long been recognized as a risk factor for coronary artery disease, myocardial infarction, and heart failure, growing evidence suggests a distinct pathophysiological role in ischemic stroke. Genetic determinants and notable racial disparities, such as higher Lp(a) levels in Africans and South Asians, along with its stable plasma concentrations throughout life, position Lp(a) as a promising biomarker for vascular risk stratification. However, despite its well-established pro-atherogenic and pro-thrombotic properties, the precise contribution of Lp(a) to different stroke subtypes remains unclear. These knowledge gaps underscore the need for a more refined approach to patient selection, considering both Lp(a) levels and the broader context of atherosclerotic burden.

The introduction of Lp(a)-lowering therapies, such as pelacarsen and olpasiran, represents a significant advancement in stroke prevention. Nonetheless, whether these agents meaningfully reduce the risk of stroke remains to be determined. Given the emerging evidence linking elevated Lp(a) levels to ICAD and CAA, further research is required to clarify its role in both ischemic and hemorrhagic strokes.

In clinical practice, neurologists should consider measuring Lp(a) levels in patients with cryptogenic stroke, ESUS, recurrent ischemic stroke despite optimal risk factor control, early-onset stroke, intracranial or extracranial atherosclerosis, and concomitant coronary artery disease. Routine screening may also be warranted in individuals with a strong family history of stroke or cardiovascular disease, particularly at a young age. Lp(a) levels, ideally measured in nmol/L, should be assessed in conjunction with clinical and imaging findings. Elevated levels (>125 nmol/L) should prompt an evaluation of modifiable non-genetic factors and consideration of Lp(a)-lowering agents. Conversely, individuals with low Lp(a) concentrations (<75 nmol/L) should undergo neuroimaging and carotid imaging to rule out Lp(a)-related pathologies, followed by repeat measurements, if necessary. While lipid-lowering therapies and risk factor modification remain the cornerstones of vascular risk reduction, targeted Lp(a)-lowering therapies should be considered for high-risk individuals with markedly elevated Lp(a) levels. Notably, PCSK9 inhibitors should be prioritized due to their dual Lp(a) and LDL-lowering effects. Future research should focus on multicenter trials to delineate the association between Lp(a) levels, stroke subtypes, and clinical outcomes to guide the development of therapeutic thresholds and evidence-based treatment strategies for stroke. Refining patient selection and integrating early detection, risk stratification, and precision therapy are essential for optimizing stroke prevention and improving patient outcomes.

## Data Availability

All data analyzed in the present study are included in this article.

## Supplementary Materials

The following supporting information can be downloaded at: https://www.mdpi.com/article/10.3390/jcm14092990/s1.

## Abbreviations

The following abbreviations are used in this manuscript:

AF	Atrial Fibrillation
Apo(a)	Apolipoprotein(a)
ApoB-100	Apolipoprotein B-100
ASOs	Antisense Oligonucleotides
ASCVD	Atherosclerotic Cardiovascular Disease
BAO	Basilar Artery Occlusion
CAA	Cerebral Amyloid Angiopathy
CAVD	Calcific Aortic Valve Disease
CCS	Canadian Cardiovascular Society
CCHS	Copenhagen City Heart Study
CGPS	Copenhagen General Population Study
CI	Confidence Interval
CRISPR-Cas9	Clustered Regularly Interspaced Short Palindromic Repeats–CRISPR-Associated Protein 9
CSF	Cerebrospinal Fluid
cSVD	Cerebral Small Vessel Disease
EAS	European Atherosclerosis Society
ECAS	Extracranial Atherosclerotic Stenosis
ESUS	Embolic Stroke of Undetermined Source
EVT	Endovascular Therapy
FDA	Food and Drug Administration
GBD	Global Burden of Disease
HR	Hazard Ratio
ICAD	Intracranial Atherosclerotic Disease
ICAS	Intracranial Atherosclerotic Stenosis
ICH	Intracerebral Hemorrhage
ICAM-1	Intracellular Adhesion Molecule-1
ISNA	In-Stent Neoatherosclerosis
KIV	Kringle IV
LAA	Large-Artery Atherosclerosis
LDL	Low-Density Lipoprotein
Lp(a)	Lipoprotein(a)
LVO	Large Vessel Occlusion
MACE	Major Adverse Cardiovascular Events
NLA	National Lipid Association
OR	Odds Ratio
OxPLs	Oxidized Phospholipids
PAI-1	Plasminogen Activator Inhibitor-1
PCSK9i	Proprotein Convertase Subtilisin/Kexin Type 9 Inhibitors
PFO	Patent Foramen Ovale
PTAS	Percutaneous Transluminal Angioplasty and Stenting
RCT	Randomized Controlled Clinical Trial
REGARDS	Reasons for Geographic And Racial Differences in Stroke
siRNA	Small Interfering RNA
SNP	Single Nucleotide Polymorphism
TIA	Transient Ischemic Attack
TF	Tissue Factor
TOAST	Trial of Org 10172 in Acute Stroke Treatment
VCAM-1	Vascular Cell Adhesion Molecule-1
VTE	Venous Thromboembolism
WSO	World Stroke Organization
