# Supplementary material for: Lipoprotein(a) as a Stroke Biomarker: Pathophysiological Pathways and Therapeutic Implications
Source: J Clin Med. 2025 Apr 25;14(9):2990. doi: 10.3390/jcm14092990 (PMC12072530; doi:10.3390/jcm14092990)

SUPPLEMENTARY MATERIAL

A. Search strategy

Search algorithm in MEDLINE (PubMed): ("lipoprotein a"[MeSH Terms] OR "lipoprotein a"[All Fields] OR "lipoprotein a"[All Fields]) AND ("atherosclerosis"[MeSH Terms] OR "atherosclerosis"[All Fields] OR "atheroscleroses"[All Fields] OR ("stroke"[MeSH Terms] OR "stroke"[All Fields] OR "strokes"[All Fields] OR "stroke s"[All Fields]) OR "cerebrovascular"[All Fields] OR ("intracranial"[All Fields] OR "intracranially"[All Fields]))

Search algorithm in Scopus: TITLE-ABS-KEY ((lipoprotein AND a) AND ((ischemic OR hemorrhagic) AND stroke))

B. PRISMA flowchart

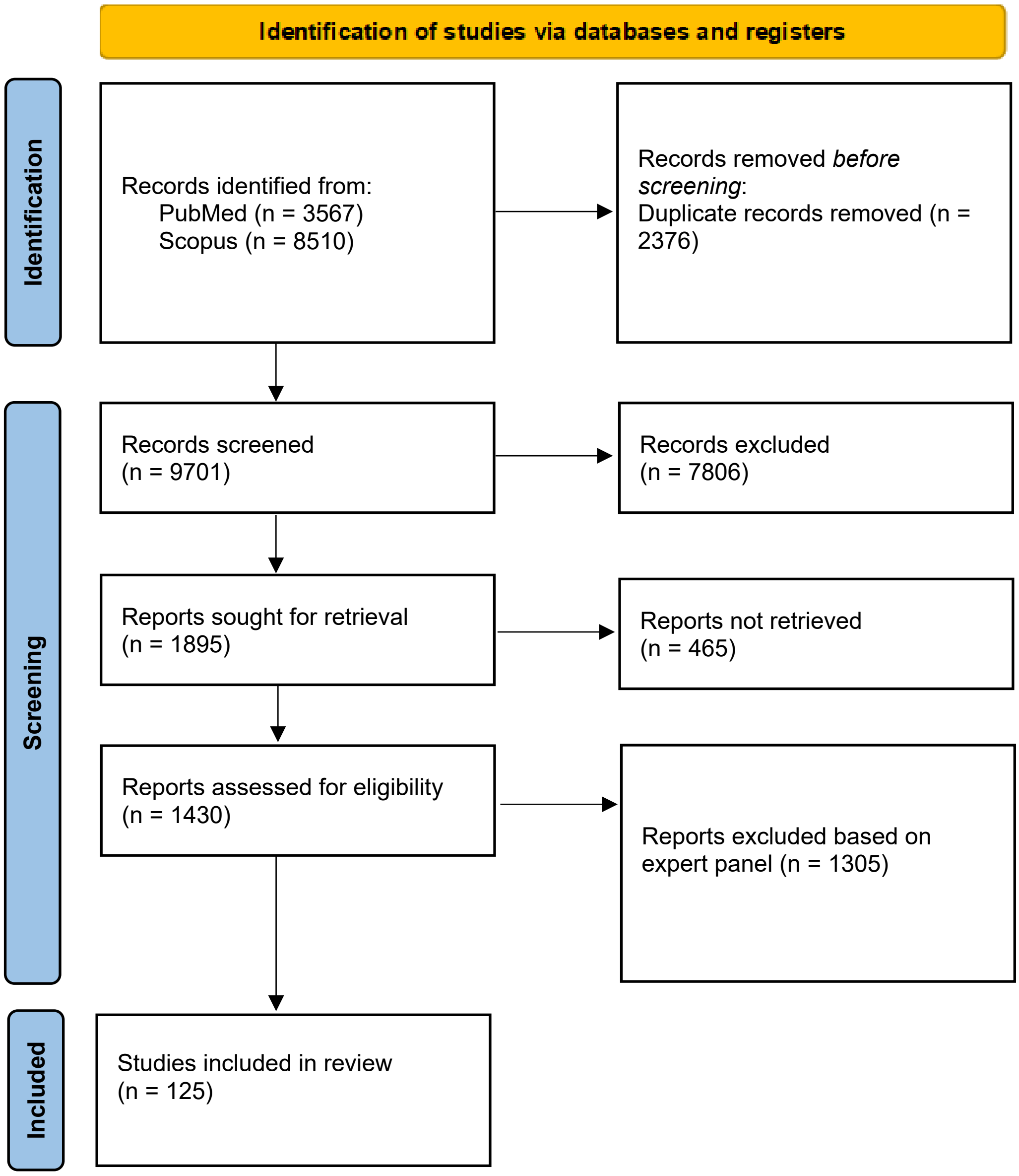

Supplement: Supplementary file 1 [file jcm-14-02990-s001.zip › jcm-3546421-supplementary.pdf]
